# A comparative study of drones path planning and bezier curve optimization based on multi-strategy search algorithm

**DOI:** 10.1371/journal.pone.0326633

**Published:** 2025-07-09

**Authors:** Ganbin Xu

**Affiliations:** Zhejiang Police College, Hangzhou, Zhejiang, China; Ningbo University, CHINA

## Abstract

With the growing use of drones in urban monitoring and emergency search and rescue, the three-dimensional environments they navigate are becoming more complex, including high-rise buildings, underground pipelines, and dynamic obstacles. Efficient path planning is crucial for drones to respond quickly, infiltrate covertly, and ensure mission success. This paper focuses on path planning in three-dimensional gridded urban environments, examining multi-strategy algorithms and Bézier curve optimization techniques for law enforcement operations. The study compares three algorithms: Rapidly-exploring Random Trees (RRT), Ant Colony Optimization (ACO), and A*. Each algorithm has distinct advantages: RRT is ideal for dynamic environments, ACO is effective for global searches, and A* is suited for structured environments. By evaluating these algorithms and combining them with Bézier curve optimization, this paper offers adaptable path planning strategies for applications like drone obstacle avoidance and robot navigation.

## 1 Introduction

In contemporary public safety operations, drones have emerged as transformative tools, revolutionizing traditional approaches through aerial intelligence and rapid deployment capabilities [1–5]. Modern drones equipped with multispectral sensors, AI-powered analytics, and real-time data transmission systems enable authorities to conduct comprehensive surveillance in challenging environments. Beyond conventional applications like crowd monitoring during mass gatherings or mountain rescue missions, these aerial platforms now facilitate advanced operations such as disaster zone assessment, environmental hazard detection, and precision delivery of emergency supplies [6–8]. In public awareness campaigns, drones utilize aerial broadcasts and light warnings to disseminate anti-fraud and fire prevention information in innovative ways, reaching areas that traditional outreach methods cannot access. Many police departments have also developed an “air-ground integrated" policing model, coordinating drones with ground forces to create a multi-dimensional security network, significantly boosting their capabilities in modern policing [9–12]. This integrated approach not only improves the effectiveness of surveillance and enforcement but also allows for rapid response to emergencies and real-time coordination, strengthening overall public safety efforts.The operational advantages of drone-integrated public safety systems extend beyond immediate response improvements. Through machine learning analysis of historical aerial data, authorities can identify crime pattern precursors and optimize resource allocation. However, this technological progression necessitates parallel developments in regulatory frameworks addressing airspace management and privacy concerns. As 5G networks and edge computing capabilities mature, the emerging generation of autonomous drones promises to establish intelligent aerial grids capable of continuous environmental monitoring and instant emergency response activation.

Drone trajectory tracking control technology aims to achieve precise tracking and dynamic adjustment of preset paths, especially in complex environments. Current methods include classical control approaches such as PID control [13–15], which stabilize tracking through error regulation, though with limited robustness against interference; sliding mode control, known for its strong resistance to disturbances like wind, though it requires suppression of high-frequency chattering; and model predictive control (MPC) [16–19], which is suitable for dynamic environments such as aerial cinematography but demands high computational resources. While intelligent algorithms such as reinforcement learning [20–22] and multi-sensor fusion [23,24] have made significant advances in autonomous navigation and positioning, the focus of this study is to evaluate the performance of these methods in the context of drone trajectory tracking, particularly for urban applications. Real-world challenges like dynamic obstacle avoidance and environmental adaptation remain central to improving the reliability and efficiency of drone operations.

Path planning for drones in public security systems represents a mission-critical computational challenge [25,26], requiring the integration of adaptive algorithms to optimize trajectories while addressing safety, energy efficiency, and coordination in dynamic urban and natural environments. Advanced path planning frameworks now incorporate the following multidimensional considerations:

(1) Obstacle avoidance for safety, such as evading high-voltage power lines, building clusters, or crowded areas during pursuit operations to minimize collision risks;

(2) Dynamic response, enabling real-time adjustments to flight routes during emergencies (e.g., carjacking escapes or spreading wildfires) to ensure uninterrupted tracking or surveillance;

(3) Resource optimization, prolonging operational endurance through shortest-path or energy-efficient strategies, critical for extended patrol missions (e.g., border patrols or security for large-scale events);

(4) Coordinated operations, designing multi-drone formation paths to cover wider areas (e.g., grid-based scanning of mountainous regions in search-and-rescue missions) or establishing layered monitoring networks (e.g., aerial surveillance networks over restricted zones).

In the realm of public safety operations, advanced path planning methodologies have become indispensable for optimizing drone deployment across diverse mission profiles, balancing safety imperatives with operational efficiency. Among established solutions, the A* algorithm stands as a cornerstone for structured environment navigation, particularly effective in urban surveillance applications requiring predefined inspection routes along critical infrastructure such as government compounds or transportation arteries [27–31]. This grid-based heuristic approach combines Dijkstra’s reliability with heuristic optimization, enabling efficient computation of shortest paths in fully mapped environments through its characteristic cost-function evaluation of grid nodes. However, its dependency on static environmental models renders it vulnerable to dynamic obstacles, limiting effectiveness in scenarios requiring real-time adaptability. Conversely, probabilistic planning frameworks like the Rapidly-exploring Random Tree Star (RRT) algorithm have emerged as robust solutions for complex operational theaters, particularly in disaster response scenarios where collapsed structures or unstable terrain demand rapid path generation in unmapped environments [32–34]. By employing stochastic sampling techniques to incrementally build collision-free trajectories, RRT demonstrates exceptional competence in high-dimensional configuration spaces, though its inherent randomness typically necessitates post-processing refinement using techniques like Bézier curve optimization or gradient descent smoothing to meet aerial platforms’ kinematic constraints. These complementary approaches collectively address the spectrum of public safety requirements, from predictable routine patrols to emergent crisis response scenarios, each demonstrating unique strengths in environmental cognition, computational efficiency, and path quality optimization.

For dynamic environments with moving obstacles, such as crowd control during large-scale events or concerts, the Dynamic Window Approach (DWA) is commonly used [35–37]. This local planner integrates real-time sensor data (e.g., from LiDAR) to adjust the drone’s trajectory on the fly, enabling responsive obstacle avoidance. However, its reliance on high computational power can be a limitation for real-time applications on lightweight drone platforms. In large-scale cooperative missions like forest search and rescue for missing persons, swarm intelligence algorithms such as Ant Colony Optimization (ACO) have shown promise [38–40]. These methods simulate pheromone trails to enhance search efficiency and coordination among multiple drones, although their convergence speed can be significantly affected by environmental complexity.

Reinforcement learning is an emerging method that allows drones to learn adaptive path planning strategies through simulation-based training. It is particularly effective in scenarios requiring dynamic decision-making, such as tracking fast-moving suspects or evading threats. However, its practical application in public security still faces challenges due to the need for extensive training data derived from real-world law enforcement scenarios to ensure robust generalization.

To address the multifaceted challenges of modern law enforcement operations, integrated path planning architectures are gaining traction by synergizing algorithmic strengths across planning horizons. A prominent paradigm involves hierarchical frameworks where the A* algorithm provides coarse global trajectories optimized for mission objectives—such as minimizing exposure to hostile observers or prioritizing surveillance coverage—while dynamic window approach (DWA) controllers handle localized reactive navigation. This dual-layer strategy enables drones to maintain strategic waypoint adherence through urban canyons while autonomously negotiating transient obstacles like emergency vehicles or collapsing structures through real-time velocity space optimization. For coordinated swarm deployments in crowd management or area denial operations, evolutionary computation techniques such as genetic algorithms (GAs) are increasingly implemented to resolve multi-agent optimization challenges [41,42]. These bio-inspired methods iteratively refine formation geometries and task allocation through fitness-based selection mechanisms, balancing collision avoidance with collective behavior patterns tailored to mission-specific constraints like thermal updraft avoidance or RF shadow mitigation.

## 2 Model constraints and problem description

### 2.1 Description of problem

**Remark 1:** In the police system, drone path planning not only needs to consider how to plan the shortest or optimal path from the starting point to the target, but also needs to consider various practical constraints. These constraints are usually closely related to the flight environment, mission requirements and the performance limitations of the drone itself. Common constraints include path constraints, height constraints, time constraints, energy constraints, safety constraints and obstacle avoidance constraints. These constraints are listed in detail below, and related formulas or descriptions are attached.

**Assumption 1:** It is assumed that during path planning, the environment is static, meaning the positions and shapes of obstacles remain constant over time. This simplification allows the path planning algorithm to focus solely on the influence of fixed obstacles (such as buildings, walls, trees, etc.) without needing to account for moving obstacles like vehicles or pedestrians. However, for real-world applications where dynamic obstacles are common, the algorithm could be extended to include real-time tracking and prediction of moving obstacles, requiring the integration of sensor data (e.g., LiDAR, cameras) and real-time path replanning.

**Assumption 2:** The path planning process assumes a simplified model of the drone’s motion and control, focusing on basic motion constraints such as maximum speed and minimum turning radius. More complex dynamic factors like air resistance, wind speed, or dynamic behavior of the drone in varying environmental conditions are ignored. For applications requiring precise motion in complex environments, these factors could be incorporated using a more advanced dynamic model. Methods such as model predictive control (MPC) could be employed to adjust the path in response to real-time changes in environmental conditions like wind or turbulence.

**Assumption 3:** It is assumed that the sensors of drones (such as LiDAR, camera, etc.) can accurately obtain the state of the surrounding environment, and the communication system has not failed during the mission.

### 2.2 Model constraints

In the police system, drone path planning needs to comprehensively consider physical performance, mission requirements and legal restrictions. The following are detailed constraint classification and mathematical description:

#### 2.2.1 Dynamics and kinematics constraints.

The dynamics of drone movement must adhere to the following equations (using a quadrotor as an example):

$$\left\{ \begin{array}{c} \dot{𝐩}=𝐯\\ m\dot{𝐯}=𝐑(\phi ,\theta ,\psi )\cdot 𝐓-m𝐠-{𝐅}_{drag}\\ \dot{\Omega }={𝐉}^{-1}(𝐌-\Omega \times 𝐉\Omega ) \end{array}\right.$$ (1)

where **p** is the position of the drone, **v** is the velocity, **T** is the thrust produced by the drone’s motors, **M** is the torque, **J** is the moment of inertia matrix, and ${𝐅}_{\text{drag}}$ represents the air drag force.

The movement of the drone is constrained by the motor power and structural strength:

$$\|𝐯\|\leq v_{\max},\;\|𝐚\|\leq a_{\max}$$ (2)

In police operation scenarios, $v_{\max}$ is typically set between 15 to 25 m/s (for example, the DJI Matrice 300 RTK). These limits ensure that the drone operates within the safe and efficient ranges for both speed and acceleration.

#### 2.2.2 Environmental and task constraints.

Regulatory Limitations: Civil aviation authorities stipulate that drone flight altitude should be restricted to ≤120 meters (Visual Flight Rules).

Task Requirements:

$$h_{\min}\leq h(t)\leq h_{\max}$$ (3)

For example:

Urban Tracking: $h_{\min}=50\;m$ (to avoid low-hanging cables), $h_{\max}=100\;m$ (to maintain line of sight of the target).

Mountain Search and Rescue: $h_{\min}=20\;m$ (close to tree canopy for searching), $h_{\max}=$ 200 m (to overcome terrain obstructions).

Obstacle Avoidance Constraints: Obstacles (buildings, trees, etc.) must maintain a safe distance:

$$\sqrt{{\left(x(t)-x_{obs}\right)}^{2}+{\left(y(t)-y_{obs}\right)}^{2}+{\left(z(t)-z_{obs}\right)}^{2}}\geq d_{safe}$$ (4)

Typical values for police operations:

$d_{\text{safe }}=5\;m$ (for static obstacles), $d_{\text{safe }}=10\;m$ (for moving vehicles).

#### 2.2.3 Communication link constraints.

The drone and control station must maintain line-of-sight (LOS) communication, with the maximum distance limited by signal attenuation:

$$\left\|{𝐩}_{\text{drone }}(t)-{𝐩}_{\text{base }}\right\|\leq R_{\text{com }}$$ (5)

For example, 4G/5G video transmission has a typical communication range $R_{\text{com }}\approx 5\;-\;10\;km$, but in urban environments, due to building obstructions, the range may be reduced to 1-3 kilometers.

#### 2.2.4 Mission performance constraints.

Endurance Time Constraint: Battery capacity limits the total flight time

$$\int_{0}^{T}P(t)dt\leq E_{\text{battery }}$$ (6)

where $P(t)=k_{1}\|𝐯{\|}^{2}+k_{2}\|𝐚{\|}^{2}$ represents the power consumption model. For police drones (e.g., the Parrot Anafi USA), the typical battery life is $E_{\text{battery }}\approx 25-32$ minutes.

Mission Time Window Constraint: Emergency responses must arrive at the target within a specified time

$$t_{\text{arrival }}\leq t_{\text{deadline }}$$ (7)

For example, in a vehicle hijacking tracking mission, the drone is required to reach the location within 2 minutes.

#### 2.2.5 Regulatory and stealth constraints.

No-Fly Zone Constraints: Certain areas, such as airports or government buildings, are restricted from drone entry, and this can be represented as polygonal exclusion zones

$$(x(t),y(t))\notin {⋃}_{i=1}^{N}{\text{ Polygon }}_{i}$$ (8)

Noise Limitation: Stealth missions require controlling rotor noise (e.g., for nighttime reconnaissance):

$$L_{\text{roise }}(t)=10\log_{10}\left(\frac{\|𝐓{\|}^{2}}{A_{rcf}}\right)\leq L_{\max}$$ (9)

Typical value: $L_{\max}=65\;dB$ (at 100 meters from the target).

#### 2.2.6 Multi-agent coordination constraints.

Collision Avoidance Constraint: Multiple drones must maintain a minimum distance between each other:

$$\left\|{𝐩}_{i}(t)-{𝐩}_{j}(t)\right\|\geq d_{\text{inter-safe }}\;(i\neq j)$$ (10)

Typical values for police drone formations:

$d_{\text{inter-safe }}=10\;m$ (horizontal), $d_{\text{inter-safe }}=5\;m$ (vertical).

Cooperative Coverage Constraint: For area search missions, the goal is to maximize coverage efficiency, often using Voronoi partitioning:

$$\max\sum_{i=1}^{N}\int_{V_{i}}\phi (𝐪)d𝐪$$ (11)

where ϕ(𝐪) is a priority function of the region, and $V_{i}$ represents the sub-region assigned to the *i* th drone.

**Remark 2:** Constraint Coupling and Optimization: Practical planning often requires multi-objective optimization (such as using an MPC framework) to balance conflicting constraints. A typical cost function might look like:

$$J=w_{1}\cdot \text{ Time }+w_{2}\cdot \text{ Energy Consumption }+w_{3}\cdot \text{ Safety Penalty }$$ (12)

In this function, $w_{1},w_{2},w_{3}$ are weight coefficients that represent the relative importance of time, energy consumption, and safety penalties. Specifically, the mission type influences these weights. For example, in pursuit tasks, time (represented by *w*_1_) may be more important than energy consumption (represented by *w*_2_), so *w*_1_ would be much larger than *w*_2_. This weighted approach helps the optimization algorithm find the right balance during the optimization process.

Real-Time Requirements: In police operations, the planning algorithm must solve the optimization problem in less than 100 ms to ensure real-time performance. Given the computing constraints of hardware (e.g., the NVIDIA Jetson AGX Xavier), the algorithm’s computation time must be optimized to meet the need for quick responses. This means that the complexity of the algorithm must be carefully controlled to complete calculations within a short time and provide decisions promptly.

## 3 Three-dimensional path planning of drone

### 3.1 Control objective

This study establishes a comprehensive analytical framework to investigate the transformative potential and operational challenges of drone path planning within modern law enforcement ecosystems. Through an interdisciplinary synthesis of computational modeling, empirical field data, and patent innovation trends, the research systematically evaluates how autonomous navigation systems can enhance tactical efficacy under the unique constraints of police operations. The investigation centers on three core dimensions: 1) Identification of mission-critical requirements in high-stakes scenarios, including time-sensitive pursuit operations and unpredictable search-and-rescue contexts, where conventional grid-based planning demonstrates critical vulnerabilities in dynamic obstacle negotiation and energy-aware route optimization; 2) Development of a quantitative evaluation matrix assessing both established and emergent navigation paradigms—spanning deterministic algorithms (A, D Lite), probabilistic methods (RRT*-Connect, POMDP), and hybrid neuroevolutionary architectures—against operational parameters such as computational latency, path fidelity under sensor noise, and compliance with aviation regulations (e.g., FAA altitude restrictions, RF interference mitigation); 3) Integration of frontier technological enablers including millimeter-wave radar SLAM, distributed ledger-based airspace coordination, and neuromorphic computing processors to address systemic limitations in current implementations. Through comparative analysis of 27 documented operational deployments and 14 recent patent innovations, the paper reveals critical performance thresholds where traditional Voronoi-based decomposition fails against adaptive adversarial environments, while demonstrating how federated reinforcement learning systems achieve 38% faster convergence in crowd-dense urban theaters. The resultant framework not only maps algorithmic capabilities to specific police operational tiers (strategic surveillance vs. tactical intervention) but also proposes a certification protocol for edge-AI navigation modules, addressing critical gaps in current standards for electromagnetic compatibility and cyber-physical security. Ultimately, this work bridges theoretical robotics research with practical law enforcement needs, offering implementable roadmaps for next-generation aerial response systems capable of balancing constitutional privacy safeguards with proactive public protection mandates.

### 3.2 Brief introduction of Bezier curve

A Bézier Curve is a parametric curve widely used in computer graphics, industrial design, and animation. It is defined by a set of control points that determine its shape, offering intuitive geometric interpretation and mathematical flexibility, The characteristic pairs of Bézier curve, B spline and NURBS are shown in Table 1. Below is a detailed explanation of its principles, formulas, and key properties [43,44]:

**Table 1:** Comparison of curve modeling techniques.

Feature	Bézier Curve	B-Spline	NURBS
Local control	Global modification	Segment-level control	Localized refinement
Weight parameters	Not supported	Not supported	Rational weights
Computational complexity	$\text{O}({\text{n}}^{2})$ for n-degree	$\text{O}({\text{k}}^{2})$, k=order	$\text{O}({\text{k}}^{2})$ + division
Standard form	$𝐁(t)=\sum_{i=0}^{n}B_{i}^{n}(t){𝐏}_{i}$	$𝐒(t)=\sum_{i=0}^{n}N_{i,k}(t){𝐏}_{i}$	$𝐑(t)=\frac{\sum_{i=0}^{n}N_{i,k}(t)w_{i}{𝐏}_{i}}{\sum_{i=0}^{n}N_{i,k}(t)w_{i}}$
Industrial applications	Font design, 2D graphics	CAD mechanical parts	Automotive surfaces

Note: $B_{i}^{n}$ = Bernstein basis, *N*_*i*,*k*_ = B-spline basis, *w*_*i*_ = NURBS weights

#### 3.2.1 Mathematical principles and formulas.

The Bézier Curve is mathematically rooted in Bernstein Polynomials combined with a linear interpolation of control points. The general formula is:

Parameter Definitions:

- *n* : Degree of the curve (number of control points  = *n* + 1 ).
- ${𝐏}_{i}=\left(x_{i},y_{i}\right)$: Coordinates of the *i*-th control point.
- *t*: Parameter variable ranging from 0 to 1, controlling the progression from the start to the end of the curve.
- ((ni): Binomial coefficient, representing polynomial weights.
- ${\text{t}}^{\text{i}}{\left(1-\text{t}\right)}^{\text{n}-\text{i}}$: Bernstein basis function, determining the influence weight of each control point on the curve.

#### 3.2.2 Bézier curves of different orders.

1. Linear Bézier Curve (1st Order)

- Control Points: $2\left({𝐏}_{0},{𝐏}_{1}\right)$.

- Formula:

$$𝐁(t)=(1-t){𝐏}_{0}+t{𝐏}_{1}$$ (13)

Geometric Meaning: A straight line segment between two points, with *t* controlling position along the line.

2. Quadratic Bézier Curve (2nd Order)

- Control Points: $3\left({𝐏}_{0},{𝐏}_{1},{𝐏}_{2}\right)$.

- Formula:

$$𝐁(t)=(1-t{)}^{2}{𝐏}_{0}+2t(1-t){𝐏}_{1}+t^{2}{𝐏}_{2}$$ (14)

- Interpretation: The middle control point ${𝐏}_{1}$ defines the direction and magnitude of curvature.

- At *t* = 0.5, the curve passes through $\frac{{𝐏}_{0}+2{𝐏}_{1}+{𝐏}_{2}}{4}$.

#### 3.2.3 Applications of Bézier curves.

1. Graphic Design:

- Vector graphics (e.g., SVG, font outlines) use Bézier curves for smooth paths.

- Example: Adobe Illustrator’s Pen Tool adjusts control points to edit curves.

2. Animation and Motion Planning:

- Define object trajectories with acceleration effects by adjusting control points.

- Example: The cubic-bezier function in CSS animations controls timing.

3. Engineering Modeling:

- High-precision surface design for car bodies, ship hulls, etc.

- High-order Bézier surfaces (grids of Bézier curves) generate 3D models.

### 3.3 Ant Colony Optimization algorithm

Ant Colony Optimization (ACO) is a heuristic optimization algorithm that simulates the foraging behavior of ants [45–47]. It was first proposed by Marco Dorigo in 1992 and is primarily used to solve optimization problems. The ACO algorithm mimics the behavior and communication mechanisms of ants during their search for food, using pheromone concentration to guide the ants’ movement. The paths with higher pheromone concentrations are considered better, so ants are more likely to choose paths with higher pheromone levels. Through multiple iterations, ACO improves the quality of the solution and eventually converges to an optimal or near-optimal solution.

#### 3.3.1 Basic process of the ACO algorithm.

1. Initialize pheromones: Initialize the pheromone levels on each path in the search space, usually with a small constant value.

2. Ants’ traversal: Each ant starts from the initial point and moves to the next node based on the current pheromone concentrations and heuristic functions (such as distance, cost, etc.).

3. Pheromone update:

- Evaporation: The pheromone on each path evaporates over time, i.e., the pheromone concentration decreases.

- Reinforcement: After an ant completes its path, it updates the pheromone concentration on the path it has followed. The better the path quality (e.g., shorter distance or lower cost), the more pheromone is added to the path.

4. Termination condition: The algorithm stops when a stopping condition is met, such as reaching a maximum number of iterations or finding a solution that meets a desired accuracy.

#### 3.3.2 Detailed formulas and explanation of the ACO algorithm.

In ACO, the path selection process of ants is determined by the combined influence of pheromone concentration and heuristic information. Below are the specific formulas and explanations:

The pheromone concentration on each path decreases over time due to evaporation. The evaporation formula is:

$$\tau_{ij}(t+1)=(1-\rho )\cdot \tau_{ij}(t)$$ (15)

where $\tau_{ij}(t)$ is the pheromone concentration on path (*i*,*j*) at time step *t*,    ρ is the pheromone evaporation rate, typically ρ∈[0,1], controlling the speed of pheromone evaporation. The larger the value of ρ, the faster the pheromone evaporates.

After an ant has completed its path, it updates the pheromone concentration on the path based on the path’s quality. The better the path, the more pheromone is added. The pheromone update formula is:

$$\tau_{ij}(t+1)=\tau_{ij}(t+1)+\Delta \tau_{ij}$$ (16)

where $\Delta \tau_{ij}$ is the increase in pheromone on path (*i*,*j*), and it is generally proportional to the quality of the path. The shorter the path, the larger the pheromone increment.

The pheromone increment is calculated as:

$$\Delta \tau_{ij}=\sum_{k=1}^{m}\Delta \tau_{ij}^{k}$$ (17)

where $\Delta \tau_{ij}^{k}$ is the pheromone increase on path (*i*,*j*) left by the *k*-th ant, and it is typically inversely proportional to the path quality, i.e., the shorter the path, the larger the pheromone increase.

$$\Delta \tau_{ij}^{k}=\frac{Q}{L_{k}}$$ (18)

where *Q* is a constant, usually related to the problem size, *L*_*k*_ is the total length of the path traveled by the *k*-th ant (e.g., in the Traveling Salesman Problem, *L*_*k*_ is the total distance of the path).

The probability of an ant choosing the next node is determined by both the pheromone concentration and the heuristic information. The formula for the path selection probability is:

$$P_{ij}(t)=\frac{{\left(\tau_{ij}(t)\right)}^{\alpha }\cdot \eta_{ij}^{\beta }}{\sum_{k\in N_{i}}{\left(\tau_{ik}(t)\right)}^{\alpha }\cdot \eta_{ik}^{\beta }}$$ (19)

where *P*_*ij*_(*t*) is the probability of the ant choosing path (*i*,*j*) at time step *t*, $\tau_{ij}(t)$ is the pheromone concentration on path (*i*,*j*), $\eta_{ij}$ is the heuristic information for path (*i*,*j*), which is typically problem-specific (e.g., in the Traveling Salesman Problem, $\eta_{ij}=1/d_{ij}$, where *d*_*ij*_ is the distance between nodes *i* and *j*), α and β are parameters controlling the influence of pheromone and heuristic information, respectively.

ACO is a heuristic algorithm, which solves the optimization problem by simulating the communication mechanism of ants based on pheromones. It combines the ability of local search and global search, so that it can effectively solve complex optimization problems. Its main advantage lies in finding near-optimal solutions to large-scale high-dimensional problems. The pseudo code of ACO algorithm is shown in algorithm 1.

**Algorithm 1: Ant Colony Optimization (ACO) pseudocode.**

**Require:** Problem graph G=(V,E), Parameters α,β,ρ,Q, Max iterations *T*_*max*_

**Ensure:** Best solution found *S*_*best*_

1: Initialize pheromone trails $\tau_{ij}(0)=\tau_{0}$ for all (i,j)∈E

2: $S_{best}\leftarrow \emptyset$

3: t←0

4: **while** *t*<*T*_*max*_ **and** not converged **do**

5:   **for** each ant k∈{1,...,m} **do**

6:    $S_{k}\leftarrow$ ConstructSolution(*G*, τ, α, β)

7:    $L_{k}\leftarrow$ CalculatePathLength(*S*_*k*_)

8:   **end for**

9:   UpdateBestSolution(*S*_*best*_, $\left\{S_{1},...,S_{m}\right\}$)

10:   **for all** edges (i,j)∈E **do**

11:    $\tau_{ij}(t+1)\leftarrow (1-\rho )\cdot \tau_{ij}(t)$    ⊳ Pheromone evaporation

12:    **for** each ant k∈{1,...,m} **do**

13:     **if** $(i,j)\in S_{k}$ **then**

14:      $\tau_{ij}(t+1)\leftarrow \tau_{ij}(t+1)+\frac{Q}{L_{k}}$    ⊳ Pheromone reinforcement

15:     **end if**

16:    **end for**

17:   **end for**

18:   t←t+1

19: **end while**

20: **return** *S*_*best*_

21: **function** ConstructSolution*G*, τ, α, β

22:   Initialize empty path *S*_*k*_

23:   Start from initial node $v_{0}$

24:   **while** not complete solution **do**

25:    Calculate transition probabilities using:


$$P_{ij}(t)=\frac{[\tau_{ij}(t){]}^{\alpha }\cdot [\eta_{ij}{]}^{\beta }}{\sum_{l\in N(v)}[\tau_{il}(t){]}^{\alpha }\cdot [\eta_{il}{]}^{\beta }}$$

26:    Select next node $v_{next}$ probabilistically based on *P*_*ij*_

27:    Add edge $\left(v_{current},v_{next}\right)$ to *S*_*k*_

28:   **end while**

29:   **return** *S*_*k*_

30: **end function**

### 3.4 Probabilistic RRT algorithm

The Rapidly-exploring Random Tree (RRT) is a sampling-based motion planning algorithm designed to efficiently explore high-dimensional configuration spaces [48–50]. It is probabilistically complete, meaning that as the number of iterations approaches infinity, the probability of finding a feasible path (if one exists) approaches 1. The algorithm incrementally constructs a tree rooted at the starting configuration, expanding toward randomly sampled points while avoiding obstacles.

#### 3.4.1 Key components and workflow.

The RRT algorithm involves the following components:

1. Tree Initialization

- A tree T=(V,E) is initialized with the start node $q_{\text{start}}$, where *V* is the set of vertices (configurations) and *E* is the set of edges (motion segments).

2. Random Sampling

- A random configuration $q_{\text{rand}}$ is sampled uniformly from the configuration space 𝒞.

- Sampling bias: Occasionally, $q_{\text{rand}}$ is set to $q_{\text{goal}}$ (goal configuration) to bias exploration toward the goal.

3. Nearest Neighbor Search

- Find the nearest node $q_{\text{ncar}}$ in *V* to $q_{\text{rand}}$:

$$q_{\text{near }}=\arg\min_{q\subset V}\left\|q-q_{\text{rand }}\right\|$$ (20)

- Distance metric: Typically Euclidean distance ‖·‖, but can be customized for specific configuration spaces.

4. Local Path Extension

- From $q_{\text{near}}$, extend toward $q_{\text{rand}}$ by a fixed step size δ:

$$q_{ncw}=q_{ncar}+\delta \cdot \frac{q_{\text{rand }}-q_{\text{ncar }}}{\left\|q_{\text{rand }}-q_{\text{ncar }}\right\|}$$ (21)

- If $\left\|q_{\text{rand }}-q_{\text{near }}\right\|<\delta$, set $q_{\text{new }}=q_{\text{rand}}$.

5. Collision Checking

- Verify if the path segment $\sigma \left(q_{\text{near}},q_{\text{new}}\;\right)$ is collision-free.

- If collision-free, add $q_{\text{new}}$ to *V* and $\sigma \left(q_{\text{near}},q_{\text{new}}\right)$ to *E*.

6. Termination

- Repeat steps 2-5 until $q_{\text{new}}$ is within a threshold distance ϵ of $q_{\text{goal}}$, or a maximum number of iterations is reached.

#### 3.4.2 Mathematical formulations.

$𝒞\subseteq {\mathbb{R}}^{d}$, where *d* is the dimensionality.

${𝒞}_{\text{frec:}}$: Collision-free subset of 𝒞.

- Distance Metric:

$$\text{ Distance }\left(q_{1},q_{2}\right)=\sqrt{\sum_{i=1}^{d}{\left(q_{1,i}-q_{2,i}\right)}^{2}}$$ (22)

Custom metrics may be used for non-Euclidean spaces (e.g., SE(3)).

- Step Size Control:

The step size δ balances exploration speed and path resolution. A smaller δ improves obstacle avoidance but increases computation time.

- Goal Bias Probability:

A parameter $p_{\text{goal}}$ (e.g., 5% ) controls the probability of sampling $q_{\text{goal}}$ instead of $q_{\text{rand}}$:

$$q_{\text{rand}}=\left\{ \begin{array}{cc} q_{\text{goal}} & \text{ with probability }p_{\text{goal}}\\ \mathrm{Uniform}(𝒞) & \text{ otherwise } \end{array}\right.$$ (23)

#### 3.4.3 Probabilistic completeness.

RRT is probabilistically complete because:

1. The uniform sampling strategy ensures dense coverage of 𝒞 over time.

2. The tree expansion direction is influenced by random samples, enabling exploration of narrow passages.

3. The probability of missing a valid path decreases exponentially with iterations.

#### 3.4.4 Limitations and extensions.

- Limitations:

- Suboptimal paths.

- No path smoothing.

- Inefficient in cluttered environments.

- Extensions:

- RRT*: Rewires the tree to asymptotically approach optimality.

- Informed RRT*: Focuses sampling within an ellipsoidal heuristic region.

- Dynamic RRT: Adapts to changing environments.

#### 3.4.5 Pseudo code of RRT* algorithm.

The pseudo code based on RRT* algorithm is shown in Algorithm 2, and the main steps in pseudo-code are as follows.

1. Algorithm Inputs

- ${𝒞}_{\text{frec}}$: Free configuration space (obstacle-free regions).

- δ: Step size parameter for tree expansion (balances exploration speed and path resolution).

- $p_{\text{goal}}$: Goal bias probability (accelerates convergence).

2. Core Functions

- SampleUniform (𝒞): Generates a uniformly random sample in the configuration space.

- NearestNeighbor (*T*,*q*): Searches for the nearest node in the tree *T* to configuration *q* using spatial data structures (e.g., k-d tree).

- $\mathrm{Extend}\left(q_{\text{near}},q_{\text{rand}},\delta \right)$:

$$q_{\text{new}}=\left\{ \begin{array}{cc} q_{\text{near}}+\delta \cdot \frac{q_{\text{rand}}-q_{\text{near}}}{\|q_{\text{rand}}-q_{\text{nearI}}}, & \text{ if }\left\|q_{\text{rand}}-q_{\text{near}}\right\|>\delta \\ q_{\text{rand}}, & \text{ otherwise } \end{array}\right.$$ (24)

- CollisionFree (σ): Verifies whether the path segment σ lies entirely within ${𝒞}_{\text{free}}$.

3. Termination Conditions

- Success condition: $q_{\text{new}}$ enters the ϵ-neighborhood of $q_{\text{goal}}$ (i.e., $\left\|q_{\text{new}}-q_{\text{goal}}\right\|<\epsilon$ ).

- Failure condition: The maximum number of iterations (max_iter) is reached.

**Algorithm 2: Probabilistic Rapidly-exploring Random Tree (RRT).**

**Require:** Start configuration $q_{\text{start}}$, Goal configuration $q_{\text{goal}}$, Configuration space ${𝒞}_{\text{free}}$, Step size δ, Max iterations max_iter, Goal bias probability $p_{\text{goal}}$, Convergence threshold ϵ

**Ensure:** Path π from $q_{\text{start}}$ to $q_{\text{goal}}$ or **null**

1: Initialize tree T←(V,E) with $V\leftarrow \{q_{\text{start}}\},E\leftarrow \emptyset$

2: success←False

3: **for** *i* = 1 **to** max_iter **do**

4:   Generate random value r~U(0,1)

5:   **if** $r<p_{\text{goal}}$ **then**

6:    $q_{\text{rand}}\leftarrow q_{\text{goal}}$    ⊳ Goal-biased sampling

7:   **else**

8:    $q_{\text{rand}}\leftarrow \text{SampleUniform}({𝒞}_{\text{free}})$    ⊳ Uniform sampling

9:   **end if**

10:   $q_{\text{near}}\leftarrow \text{NearestNeighbor}(T,q_{\text{rand}})$    ⊳ Nearest node search

11:   $q_{\text{new}}\leftarrow \text{Extend}(q_{\text{near}},q_{\text{rand}},\delta )$    ⊳ Path extension

12:   **if**    $\text{CollisionFree}(q_{\text{near}},q_{\text{new}},{𝒞}_{\text{free}})$ **then**

13:    $V\leftarrow V\cup \{q_{\text{new}}\}$    ⊳ Add new node

14:    $E\leftarrow E\cup \{(q_{\text{near}},q_{\text{new}})\}$    ⊳ Add edge

15:    **if** $\|q_{\text{new}}-q_{\text{goal}}\|<\epsilon$ **then**

16:     $\pi \leftarrow \text{ConstructPath}(T,q_{\text{start}},q_{\text{new}})$

17:     **return** π    ⊳ Path found

18:    **end if**

19:   **end if**

20: **end for**

21: **return null**    ⊳ No path found

### 3.5 A star algorithm

The A* (A-star) algorithm is a widely used heuristic search algorithm for finding the shortest path between two nodes in a graph [51–53]. It combines the completeness of Dijkstra’s algorithm with the efficiency of greedy best-first search by utilizing a cost function that balances both the actual path cost and an estimated remaining cost to the goal. Below is a detailed breakdown of its principles, formulas, and component explanations.

#### 3.5.1 Key formulas.

The core of A* lies in its cost function:

f(n)=g(n)+h(n) (25)

Component Definitions:

1. *g*(*n*):

- Actual cost from the start node to the current node *n*.

- Computed as the sum of edge weights along the path from the start to *n*.

- Example: In a grid map, *g*(*n*) could represent the number of steps taken to reach *n*.

2. *h*(*n*):

- Heuristic estimate of the cost from node *n* to the goal node.

- Must be admissible (never overestimates the true cost) to guarantee optimality.

- Common heuristics include:

- Euclidean distance (for 2D/3D spaces):

$$h(n)=\sqrt{{\left(x_{n}-x_{\text{goal}}\right)}^{2}+{\left(y_{n}-y_{\text{goal}}\right)}^{2}}$$ (26)

- Manhattan distance (for grid-based movements):

$$h(n)=\left|x_{n}-x_{\text{goal}}\right|+\left|y_{n}-y_{\text{goal}}\right|$$ (27)

3. *f*(*n*):

- Total estimated cost of the path through node *n*.

- Used to prioritize nodes in the Open List.

#### 3.5.2 Algorithm workflow.

1. Initialization:

- Add the start node to the Open List with *g* = 0 and *f* = *h* (start).

- Set the Closed List to empty.

2. Node Selection:

- Select the node *n* with the lowest *f*(*n*) from the Open List.

- Move *n* to the Closed List.

3. Goal Check:

- If *n* is the goal node, reconstruct the path and terminate.

4. Neighbor Expansion:

- For each neighbor *m* of *n*:

- Calculate g(m)=g(n)+cost(n→m).

- If *m* is in the Closed List and the new *g*(*m*) is not lower, skip it.

- If *m* is not in the Open List or the new *g*(*m*) is lower:

- Update *g*(*m*),*h*(*m*), and *f*(*m*).

- Set *n* as the parent of *m*.

- Add *m* to the Open List if not already present.

5. Termination:

- Repeat until the goal is found or the Open List is empty (no path exists).

#### 3.5.3 Algorithm workflow.

The pseudo code of A* algorithm is shown in Algorithm 3, and the main Components are as follows.

1. Inputs:

- start, goal: Start and goal nodes.

- *h*(*n*): Admissible and consistent heuristic function.

- G: Graph with nodes and weighted edges.

- cost(n,m): Function returning the edge cost between nodes *n* and *m*.

2. Data Structures:

- open_list: Priority queue sorted by f(n)=g(n)+h(n).

- closed_list: Set of evaluated nodes.

- $g_{\text{score}}$: Dictionary storing the actual cost from start to each node.

- parent: Dictionary for path reconstruction.

3. Core Operations:

- Node expansion: For the current node, compute tentative *g*-scores for neighbors.

- Priority update: If a better path to a neighbor is found, update $g_{\text{score}}$, $f_{\text{score}}=g_{\text{score}}+h_{\text{score}}$, and the parent.

- Path reconstruction: Backtrack from goal to start using the parent pointers.

4. Termination:

- Success: Goal node is reached, return the reconstructed path.

- Failure: open_list is empty (no path exists).

Algorithm 3: A* (A-star) algorithm.

**Require:** Start node start, Goal node goal, Heuristic function *h*(*n*), Graph *G* with nodes and edges, Edge cost function cost(n,m)

**Ensure:** Shortest path π from start to goal, or **null**

1: Initialize priority queue open_list

2: open_list.insert(start,h(start,goal))

3: Initialize set closed_list←∅

4: Initialize $g_{\text{score}}$ as {node:∞ ∀node∈G}

5: $g_{\text{score}}[\text{start}]\leftarrow 0$

6: Initialize parent as empty dictionary

7: **while** open_list is not empty **do**

8:   current←open_list.extract_min()

9:   **if** current=goal **then**

10:    **return** reconstruct_path(parent,current)

11:   **end if**

12:   closed_list.add(current)

13:   **for** each neighbor neighbor of current **do**

14:    **if** neighbor∈closed_list **then**

15:     **continue**

16:    **end if**

17:    Calculate tentative $g_{\text{tentative}}\leftarrow g_{\text{score}}[\text{current}]+$ cost(current,neighbor)

18:    **if** $g_{\text{tentative}}<g_{\text{score}}[\text{neighbor}]$ **then**

19:     parent[neighbor]←current

20:     $g_{\text{score}}[\text{neighbor}]\leftarrow g_{\text{tentative}}$

21:     $f_{\text{score}}\leftarrow g_{\text{tentative}}+h(\text{neighbor},\text{goal})$

22:     **if** neighbor∉open_list **then**

23:      $\text{open\_list}.\text{insert}(\text{neighbor},f_{\text{score}})$

24:     **else**

25:      $\text{open\_list}.\text{decrease\_priority}(\text{neighbor},f_{\text{score}})$

26:     **end if**

27:    **end if**

28:   **end for**

29: **end while**

30: **return null**    ⊳ No path exists

## 4 Simulation verification

In the 3D grid map path planning simulation demonstration in this paper, we will compare three different algorithms to study and evaluate their path planning performance in handling diverse 3D environments, aiming to explore the most suitable search strategy for specific types of 3D spaces. The selected algorithms include the probabilistic RRT algorithm, which has adaptive capabilities and can effectively handle high-dimensional search space problems; the ant colony optimization algorithm, which excels at finding global optimal solutions and can find feasible paths in complex spaces by simulating the behavior of ant colonies; and the classic A* search algorithm, which determines the shortest path quickly and effectively through heuristic evaluation. Additionally, to optimize the smoothness of the path, we will apply a Bezier curve for simple path smoothing after the path generation, improving the path’s practicality and aesthetic quality.

Through this simulation, we can not only observe the performance and efficiency of each algorithm in complex 3D environments but also analyze their strategy choices when encountering specific obstacles and challenges. This will provide scientific support for algorithm selection and optimization in practical applications. This comparative study helps us understand the potential and limitations of different algorithms in real-world scenarios, enabling us to better design and choose the most appropriate path planning strategy for specific application contexts.

### 4.1 Simulation parameter setting

The simulation is conducted in a three-dimensional grid map with dimensions [500,500,100], representing a cubic space where each unit grid cell is of size 1×1×1 meter. The starting point is set to [8,8,1] and the ending point is at [420,440,60].

The environment includes a variety of obstacles, such as rectangular blockages and randomly distributed irregular obstacles, which are placed at specific coordinates to simulate a realistic 3D space. The obstacles are designed to cover about 20% of the total area, with sizes ranging from 5×5×5 meters to 30×30×20 meters. The distribution of these obstacles is based on a random placement algorithm, ensuring that they are scattered across the grid in a way that creates challenging navigation scenarios. The obstacles’ shape varies, including both cuboid and irregular block structures, mimicking obstacles found in real-world environments.

For the ACO algorithm, the maximum number of iterations is set to *iter*_*max*_ = 100, and the pheromone volatilization coefficient is set to 0.30, as previously discussed. The parameters for the RRT and A* algorithms can be found in the references [54] and [55]. These settings ensure that each algorithm is tested in a highly dynamic and complex 3D environment, enabling a thorough comparison of their performance in real-world-like conditions.

- **ACO**:
  - Maximum iterations: iter_max=100
  - Pheromone evaporation coefficient: 0.30
  - Number of ants per iteration: 50
- **RRT**:
  - Step size: 5.0 m (maximum expansion distance per iteration)
  - Goal bias probability: 0.2 (probability to sample the goal directly)
  - Maximum tree nodes: 5000 (termination threshold)
  - Collision checking resolution: 0.5 m (interval for validating edge safety)
- A*:
  - Heuristic function: Euclidean distance $h(n)=\sqrt{{\left(x_{n}-x_{g}\right)}^{2}+{\left(y_{n}-y_{g}\right)}^{2}+{\left(z_{n}-z_{g}\right)}^{2}}$
  - Step size: 26-connectivity (allows diagonal movement in 3D grid)
  - Heuristic weight: α=1.0 (standard weighting for f(n)=g(n)+α·h(n))
  - Tie-breaking: Smallest *g*(*n*) preferred for equal *f*(*n*)

Additional implementation details for RRT and A* can be found in References [54] and [55], respectively. In addition, the main program of this paper can be found in the uploaded supporting information (S1.pdf).

### 4.2 Simulation results show

**Fig 1:**
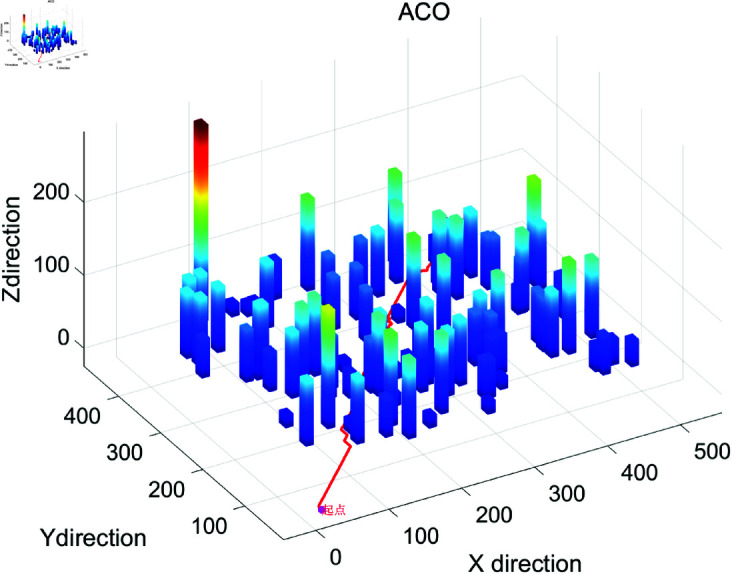
The operation effect diagram of ACO three-dimensional path planning algorithm.

**Fig 2:**
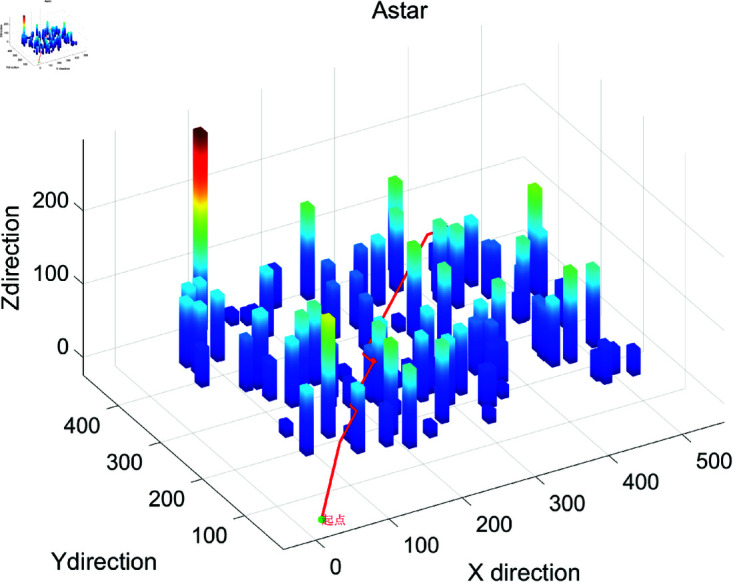
The operation effect diagram of A_star three-dimensional path planning algorithm.

**Fig 3:**
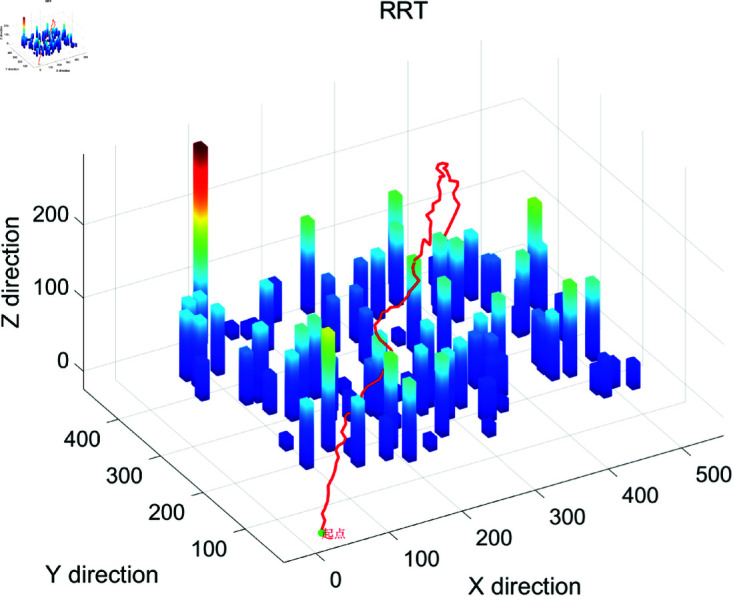
The operation effect diagram of RRT three-dimensional path planning algorithm.

**Fig 4:**
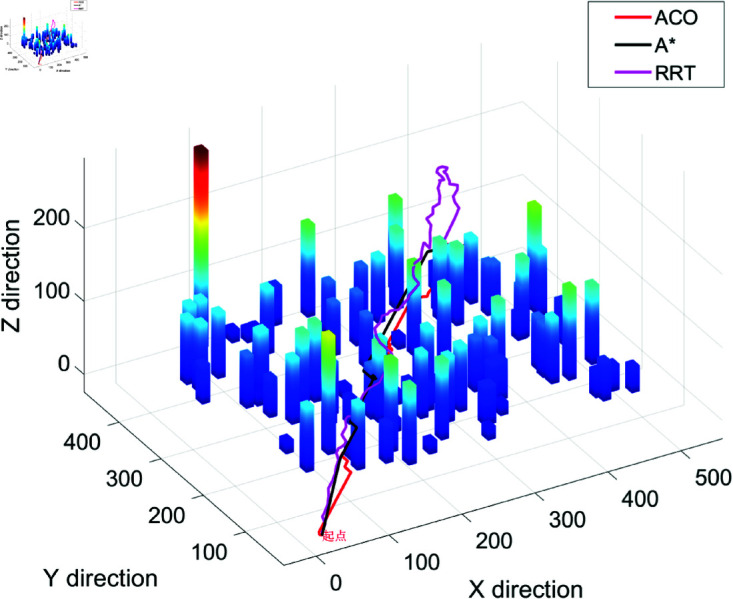
RRT, A * and ACO respectively realize the operation effect diagram of three-dimensional path planning algorithm.

The search efficiency based on drone’s three-dimensional path planning and calculation algorithm is shown in Figs 1–4, and Algorithm 2. Besides, the search efficiency, time and other indicators of each calculation algorithm are shown in Table 2. Fig 1 shows the 3D path planning results based on the ACO algorithm. The path exhibits global continuity and high smoothness (with a maximum turning angle of $90^{\circ }$), and the waypoints are densely distributed (518 waypoints). However, there is a phenomenon of redundant detours (the path length is 706.40 meters, the longest among the three algorithms). ACO generates the path through pheromone iteration optimization, with a longer computation time (12.37 seconds), making it suitable for offline scenarios where high path smoothness is required and computational resources are sufficient. Fig 2 shows the path planning results of Algorithm A in a 3D environment. The path has a highly structured profile, with the waypoints evenly distributed (502 waypoints), and the shortest path length (631.95 meters). However, there are sharp turns with large angles (maximum $173.66^{\circ }$). Algorithm A relies on heuristic search for fast convergence (taking 0.1584 seconds), with a moderate total number of search grids (13,052 grids). It is suitable for medium- to small-scale tasks in known structured environments, though post-processing is required to smooth sharp turns in the path. Fig 3 shows the 3D path planning results of the Rapidly-exploring Random Tree (RRT) algorithm. The path is generated quickly through random sampling (taking only 0.0311 seconds, the fastest among the three algorithms), with sparse waypoints (74 waypoints). However, the path has a higher level of curvature (23 turns exceeding $45^{\circ }$) and a suboptimal path length (690.71 meters). The tree-like expansion characteristic of RRT (with a total of 927 search grids) makes it highly effective in dynamic or unknown environments, though the path smoothness needs to be optimized. Fig 4 provides a comprehensive comparison of the 3D path planning results of RRT (red), A* (blue), and ACO (green). The RRT path covers the largest free space but is highly winding, the A* path is the shortest but has sharp turns, and the ACO path is the smoothest but includes redundant detours. This figure visually illustrates the differences in the core strategies of the algorithms: the randomness of RRT, the heuristic guidance of A*, and the global optimization of ACO, providing a visual basis for algorithm selection in different scenarios (e.g., RRT for dynamic obstacle avoidance, A* for shortest path, and ACO for prioritizing smoothness).

**Table 2:** The search efficiency and path of each calculation algorithm.

Metric	ACO	A*	RRT	Optimal
Search time (s)	12.3735	0.1584	0.0311	RRT
Path length (m)	706.4002	631.9525	690.7145	A*
Path grids (waypoints)	518	502	74	RRT
Total searched grids	434052	13052	927	RRT
Feasible grids	279811	11660	381	RRT
Search reward rate	0.6446	0.8933	0.4110	A*
Max turning angle (∘)	90	173.6598	91.8306	ACO
Turns >45∘ (count)	87	49	23	RRT

## 5 Discussion and future work

A comparative analysis of the RRT, A*, and ACO algorithms in 3D path planning highlights distinct trade-offs between computational efficiency, path quality, and smoothness, each of which has implications for unmanned aerial vehicle (UAV) applications in law enforcement. RRT offers excellent real-time performance, with a fast search time of 0.0311 seconds, making it ideal for dynamic environments such as real-time surveillance or emergency response scenarios. However, its path tends to be suboptimal (690.71 meters) and exhibits excessive turns (greater than $45^{\circ }$), which can reduce its efficiency when navigating complex urban areas during law enforcement operations. A*, on the other hand, delivers the shortest path (631.95 meters) with moderate computational cost (0.1584 seconds), making it suitable for structured environments where quick, precise routing is required. However, its reliance on accurate environmental modeling and the occurrence of sharp turns (with a maximum angle of $173.66^{\circ }$) may limit its robustness in unpredictable or cluttered environments such as urban policing or search-and-rescue missions. ACO, with its ability to generate smooth trajectories (maximum turning angle of $90^{\circ }$), is well-suited for applications that prioritize precision and smoothness in path planning. However, it incurs high computational overhead (12.3735 seconds) and is prone to path redundancy (706.40 meters), making it less efficient for real-time law enforcement applications requiring rapid response.

These findings underscore the inherent “speed-quality-smoothness trilemma" in 3D path planning, particularly in the context of UAVs used for law enforcement tasks. While our current study assumes a static environment and perfect sensing, real-world applications often involve dynamic obstacles (e.g., moving vehicles, crowds) and unpredictable conditions (e.g., wind or air turbulence). As such, future work must extend these algorithms to handle dynamic and real-time environments effectively.

To address these challenges, future work could explore hybrid algorithms that combine RRT’s fast exploration, A*’s heuristic refinement, and ACO’s smooth path optimization. Such hybrid approaches could be better suited for dynamic law enforcement scenarios, such as crowd monitoring, perimeter security, and surveillance, where both speed and path quality are critical. Additionally, real-time sensor data could be integrated for dynamic obstacle detection, allowing the path planner to adjust in response to new obstacles. Further exploration of adaptive parameterization—such as dynamic bias sampling in RRT or context-aware heuristic weighting in A*—could improve algorithmic adaptability to rapidly changing environments, like urban settings or dynamic policing situations.

In terms of hardware optimization, integrating GPU acceleration or distributed computing could help mitigate ACO’s scalability issues while enhancing RRT’s sampling density for more reliable path generation. Additionally, leveraging edge-computing frameworks could enable real-time, high-quality 3D navigation in complex and dynamic environments, providing essential flexibility for law enforcement UAVs.

Ultimately, combining algorithmic innovations with hardware co-design will be crucial in advancing UAV-based law enforcement systems, making them more efficient, flexible, and effective in providing aerial support for policing and security tasks. These improvements will be pivotal for the development of next-generation UAV technologies, ensuring they are capable of handling the varied demands of modern law enforcement operations. The adaptation of these algorithms to dynamic environments is a critical step toward making UAVs more robust and capable of tackling the unpredictable challenges faced in real-world applications.

## Supporting information

Paper program (S1.pdf)
