# Supplementary material for: A comparative study of drones path planning and bezier curve optimization based on multi-strategy search algorithm
Source: PLoS One. 2025 Jul 9;20(7):e0326633. doi: 10.1371/journal.pone.0326633 (PMC12240380; doi:10.1371/journal.pone.0326633)
Supplement: Paper program [file pone.0326633.s001.pdf]

MATLAB

```
clc; clear; close all

% Load function paths

addpath(genpath('./ACO3D'))

addpath(genpath('./Astar3D'))

addpath(genpath('./RRT3D'))

addpath(genpath('./Evaluation'))


Algorithm_name = {'ACO', 'Astar', 'RRT'};

% Map scale is set in the Makemap3D function

map = Makemap3D; % Map

source = [8 8 1]; % Starting point

goal = [420 440 60]; % Goal point

max_item = 800; % Maximum number of iterations

comparative_data = {}; % Record data for comparison

Global_data = {};

Straight_distance = sqrt(sum((source-goal).^2, 2)); % Straight-line distance

fprintf('Straight-line distance from the start point to the goal: \n%d\n\n',
Straight_distance);

Global_data(1, end + 1) = {num2str(source)};

Global_data(1, end + 1) = {num2str(goal)};

Global_data(1, end + 1) = {Straight_distance};

t1 = clock;


%% ******** Ant Colony Optimization (ACO) **********

figure(1)

plot3DMap(map);
```

```
text(source(1), source(2), source(3), 'Start', 'color', 'r');

text(goal(1), goal(2), goal(3), 'Goal', 'color', 'r');

scatter3(source(1), source(2), source(3), "filled", "g");

scatter3(goal(1), goal(2), goal(3), "filled", "b");

title('ACO');

popNum = 10; % Ant colony size

tic

[aco_path, aco_cost, aco_Number_of_searches, aco_Number_of_successful_searches,
aco_Number_of_failed_searches] = aco(source, goal, map, popNum); % ACO main
function

aco_time = toc;

fprintf('ACO Time: %0.3f seconds\n', aco_time);

hold on

plot3(aco_path(:, 1), aco_path(:, 2), aco_path(:, 3), 'LineWidth', 2, 'color', 'r');

view(-30, 30);

fprintf('ACO Path Length: %d \n\n', aco_cost);

[aco_max_turning_angle, aco_turning_num, aco_index] = Max_turning_angle(aco_path,
1);

comparative_data(1, end + 1) = {aco_time};

comparative_data(2, end) = {aco_cost};

comparative_data(3, end) = {size(aco_path, 1)};

comparative_data(4, end) = {aco_Number_of_searches};

comparative_data(5, end) = {aco_Number_of_successful_searches};

comparative_data(6, end) = {aco_Number_of_successful_searches /
aco_Number_of_searches};

comparative_data(7, end) = {aco_max_turning_angle};

comparative_data(8, end) = {aco_turning_num};
```

```
%% ******** A* Algorithm **********

figure(2)

plot3DMap(map);

text(source(1), source(2), source(3), 'Start', 'color', 'r');

text(goal(1), goal(2), goal(3), 'Goal', 'color', 'r');

scatter3(source(1), source(2), source(3), "filled", "g");

scatter3(goal(1), goal(2), goal(3), "filled", "b");

title('Astar');

tic % Calculate runtime

[astar_path, astar_cost, astar_Number_of_searches,
astar_Number_of_successful_searches, astar_Number_of_failed_searches] =
Astar_main(source, goal, map, max_item); % A* main function

astar_time = toc;

fprintf('Astar Time: %0.3f seconds\n', astar_time);

plot3(astar_path(:, 1), astar_path(:, 2), astar_path(:, 3), 'LineWidth', 2, 'color', 'r');

view(-30, 30);

fprintf('Astar Path Length: %d\n\n', astar_cost);

[astar_max_turning_angle, astar_turning_num, astar_index] =
Max_turning_angle(astar_path, 1);

% Record data for display

comparative_data(1, end + 1) = {astar_time};

comparative_data(2, end) = {astar_cost};

comparative_data(3, end) = {size(astar_path, 1)};

comparative_data(4, end) = {astar_Number_of_searches};

comparative_data(5, end) = {astar_Number_of_successful_searches};

comparative_data(6, end) = {astar_Number_of_successful_searches /
astar_Number_of_searches};

comparative_data(7, end) = {astar_max_turning_angle};
```

```
comparative_data(8, end) = {astar_turning_num};

%% ******** Rapidly-exploring Random Tree (RRT) **********
% subplot(1,3,3);
figure(3)
plot3DMap(map);
text(source(1), source(2), source(3), 'Start', 'color', 'r');
text(goal(1), goal(2), goal(3), 'Goal', 'color', 'r');
scatter3(source(1), source(2), source(3), "filled", "g");
scatter3(goal(1), goal(2), goal(3), "filled", "b");
title('RRT');
step = 10; % Set step size
tic
[rrt_path, rrt_cost, rrt_Number_of_searches, rrt_Number_of_successful_searches,
rrt_Number_of_failed_searches] = RRT_main(source, goal, map, step); % RRT main
function
rrt_time = toc;
fprintf('RRT Time: %0.3f seconds\n', rrt_time);
plot3(rrt_path(:, 1), rrt_path(:, 2), rrt_path(:, 3), 'LineWidth', 2, 'color', 'r');
view(-30, 30);
fprintf('RRT Path Length: %d \n\n', rrt_cost);
[rrt_max_turning_angle, rrt_turning_num, rrt_index] = Max_turning_angle(rrt_path, 1);
% Record search coverage data
comparative_data(1, end + 1) = {toc};
comparative_data(2, end) = {rrt_cost};
comparative_data(3, end) = {size(rrt_path, 1)};
comparative_data(4, end) = {rrt_Number_of_searches};
```

```
comparative_data(5, end) = {rrt_Number_of_successful_searches};

comparative_data(6, end) = {rrt_Number_of_successful_searches /
rrt_Number_of_searches};

comparative_data(7, end) = {rrt_max_turning_angle};

comparative_data(8, end) = {rrt_turning_num};


%% Print total runtime

t2 = clock;

fprintf('Total program runtime: %0.3f seconds\n\n', etime(t2, t1));


% Calculate the best option, where the displayed table order is ACO, Astar, RRT

comparative_data(1, end + 1) = {Algorithm_name{Min_value(comparative_data{1, 1},
comparative_data{1, 2}, comparative_data{1, 3})}};

for i = 2:size(comparative_data, 1)

    % Find the minimum value for each row

    comparative_data(i, end) = {Algorithm_name{Min_value(comparative_data{i, 1},
comparative_data{i, 2}, comparative_data{i, 3})}};

end

% Find the maximum value

comparative_data(6, end) = {Algorithm_name{Max_value(comparative_data{6, 1},
comparative_data{6, 2}, comparative_data{6, 3})}};


% Display the comparison results

Show_Comparative_result(Global_data, comparative_data)


% Display the three algorithms in one figure

figure(4)

plot3DMap(map);
```

```
text(source(1), source(2), source(3), 'Start', 'color', 'r');

text(goal(1), goal(2), goal(3), 'Goal', 'color', 'r');

h1 = plot3(aco_path(:, 1), aco_path(:, 2), aco_path(:, 3), 'LineWidth', 2, 'color', 'r');

h2 = plot3(astar_path(:, 1), astar_path(:, 2), astar_path(:, 3), 'LineWidth', 2, 'color', 'k');

h3 = plot3(rrt_path(:, 1), rrt_path(:, 2), rrt_path(:, 3), 'LineWidth', 2, 'color', 'm');

legend([h1, h2, h3], 'Ant Colony', 'A*', 'RRT')

%legend("boxoff")

view(-30, 30);


%%%%%%%%%%%%%%%%%%%%%%%%%%%%%%%%%%%%%%%%%%%%%%%    ACO

function [path, aco_cost, Number_of_searches, Number_of_successful_searches, Number_of_failed_searches] = aco(point1, point2, mapdata, popNum)

PopNum = popNum;       % Population size

BestFitness = [];         % Best individual

iter_max = 100; % Number of iterations

[x_max, y_max, z_max] = size(mapdata); % Get the map size


%% Pheromone initialization

pheromone = ones(x_max, y_max, z_max);

pheromone = initial_pheromone(pheromone, point2, mapdata);

Number_of_searches = 0;

Number_of_successful_searches = 0;

Number_of_failed_searches = 0;


% Initialize search paths

[flag, judges, paths, pheromone, number_of_searches, number_of_successful_searches, number_of_failed_searches] = searchpath(PopNum, pheromone, point1, point2, mapdata);
```

```
% Calculate the number of grid cells searched
Number_of_searches = Number_of_searches + number_of_searches;
Number_of_successful_searches = Number_of_successful_searches + 
number_of_successful_searches;
Number_of_failed_searches = Number_of_failed_searches + number_of_failed_searches;

fitness = CacuFit(judges, paths, PopNum);                    % Fitness calculation
[bestfitness, bestindex] = min(fitness);                    % Best fitness
bestpath = paths{1, bestindex};                              % Best path
%[worstfitness, worstindex] = max(fitness);              % Worst fitness
%worstpath = paths{1, worstindex};                         % Worst path
BestFitness = [BestFitness; bestfitness];                 % Record fitness values

%% Pheromone update
rou = 0.3; % Pheromone decay coefficient
cfit = 200 / bestfitness; % Pheromone increment
[n, m] = size(bestpath);
for i = 1:n
    % Update the pheromone at the corresponding grid cell
    pheromone(bestpath(i, 1), bestpath(i, 2), bestpath(i, 3)) = (1 - rou) * 
pheromone(bestpath(i, 1), bestpath(i, 2), bestpath(i, 3)) + rou * cfit;
end

maxpathcost = [];
%% Loop to find the optimal path
for iter = 1:iter_max
    %% Path search
```

```
    Number_of_searches = Number_of_searches + 1;
    if flag == 1
        break;
    end
    [flag, judges, paths, pheromone, number_of_searches,
number_of_successful_searches, number_of_failed_searches] = searchpath(PopNum,
pheromone, point1, point2, mapdata);
    Number_of_searches = Number_of_searches + number_of_searches;
    Number_of_successful_searches = Number_of_successful_searches +
number_of_successful_searches;
    Number_of_failed_searches = Number_of_failed_searches +
number_of_failed_searches;

    %% Fitness value calculation update
    fitness = CacuFit(judges, paths, PopNum); % Smaller fitness values are better
    [newbestfitness, newbestindex] = min(fitness);

    if newbestfitness < bestfitness
        bestfitness = newbestfitness;
        bestpath = paths{1, newbestindex};
    end

    %% Update pheromone
    cfit = 200 / bestfitness;
    [n, m] = size(bestpath);
    for i = 1:n
        pheromone(bestpath(i, 1), bestpath(i, 2), bestpath(i, 3)) = (1 - rou) *
pheromone(bestpath(i, 1), bestpath(i, 2), bestpath(i, 3)) + rou * cfit;
```

```matlab
    end

end % for loop

% Best path
path = bestpath;

% Calculate path length
pathLength = 0;
for i = 1:length(bestpath(:, 1)) - 1
    pathLength = pathLength + distance(bestpath(i, 1), bestpath(i, 2), bestpath(i, 3), bestpath(i + 1, 1), bestpath(i + 1, 2), bestpath(i + 1, 3));
end
aco_cost = pathLength;



%% Define basic data, including map dimensions, starting point, etc.
function [astar_path, astar_cost, Number_of_searches, Number_of_successful_searches, Number_of_failed_searches] = Astar_main(source, goal, map, max_item)
% Number_of_failed_searches is not used

% Number_of_searches ;% Record the total number of grids searched: successful + failed
dy_SearchArea =[];   % Dynamically plot the search area
sideMAX = 1; % Weight of h when calculating f
Max_item = max_item; % Maximum number of iterations
Number_of_searches = 0; % Record the total number of grids searched: successful + failed
Number_of_successful_searches = 0; % Number of successful searches
```

Number_of_failed_searches = 0; % Number of failed searches

%% Initialize openList and closeList

% Initially, openList only contains the start point, and closeList is empty

% openList (n*6) stores the position information, G, H, and F values of the points

openList = [source, 0, 0, 0];

% closeList stores position information and distance weight F values

closeList = [];

%% Initialize path, which is the path matrix (n*2) from the start point to any point on the map ***************************

% For the start point, its path is known, so the start point path is written

path = source;

%% ****** A* Algorithm ***************************

item = 0; % Run counter

while item <= Max_item

    % sfprintf('Iteration %d\n',item);

    item = item + 1;

    % 1. Search for the node with the smallest movement cost, and return its row number. The min function returns [value, position]

    [~, idxNode] = min(openList(:, 6)); % f value is in the sixth column of openList

    node = openList(idxNode, 1:3); % The node with the smallest cost is selected as the parent node

    % 2. Check if the goal point has been reached

    if isequal(node, goal)

        break

    end

    % ****** 3. Select the node with the smallest F value in openList as the parent node

    % Return all feasible child nodes of the parent node

    nextNodes = Astat_NextNode(map, closeList, node);

```
Number_of_searches = Number_of_searches + 26; % Total number of searches
% ******* 4. Check the child nodes around the parent node, and add or update the child nodes in openList
for i = 1:size(nextNodes, 1)
    nextNode = nextNodes(i, :);
    Number_of_successful_searches = Number_of_successful_searches + 1;
    % Calculate the cost function
    rowNode = node(1); colNode = node(2); heightNode = node(3);
    row_nextNode = nextNode(1); col_nextNode = nextNode(2); height_nextNode = nextNode(3);
    row_goalPos = goal(1); col_goalPos = goal(2); height_goalPos = goal(3);
    % Original g value + Euclidean norm (distance)
    g = openList(idxNode, 4) + norm([rowNode, colNode, heightNode] - [row_nextNode, col_nextNode, height_nextNode]);
    % Calculate h
    h = (abs(row_goalPos - row_nextNode) + abs(col_goalPos - col_nextNode) + abs(height_goalPos - height_nextNode));
    % Calculate f
    f = g + sideMAX * h;

    % Check if the child node exists in openList
    [inOpen, idx_nextNode] = check_isnumber(nextNode, openList);
    % ****** If it exists, compare the F value and update F, G, and H along with the path
    if inOpen && f < openList(idx_nextNode, 6)
        openList(idx_nextNode, 4) = g;
        openList(idx_nextNode, 5) = h;
        openList(idx_nextNode, 6) = f;
```

```
            % For the sake of plotting, swap x and y coordinates just like in the
Astat_NextNode function
            path = [path; nextNode(1), nextNode(2), nextNode(3)];
        end
        % ******* If it doesn't exist, add it to the openList
        if ~inOpen
            openList(end + 1, :) = [nextNode, g, h, f];
            path = [path; nextNode(1), nextNode(2), nextNode(3)];
        end
        dy_SearchArea = [dy_SearchArea; nextNode];
    end

    % Remove the parent node from openList and add it to closeList
    closeList(end + 1, :) = [openList(idxNode, 1:3), openList(idxNode, 6)];
    openList(idxNode, :) = [];
    % Plot the real-time path
    % plot3(closeList(:, 1), closeList(:, 2), closeList(:, 3), 'LineWidth', 1, 'color', 'r');
    % pause(0.01); % Pause to observe the process

end
%% Return the results

% Calculate the path length
% pathLength =
```
